# Leisure-time physical activity and low back pain in Switzerland: results from the 2022 cross-sectional Swiss Health Survey

**DOI:** 10.1186/s12891-026-10099-2

**Published:** 2026-06-30

**Authors:** Alexander P Schurz, Marcel Zwahlen, Jan Taeymans, Nathanael Lutz

**Affiliations:** 1 Division of Physiotherapy, Department of Health Professions, Bern University of Applied Sciences, Bern, Bern, Switzerland; 2 Department of Movement and Sport Sciences, Faculty of Physical Education and Physiotherapy, Vrije Universiteit Brussel, Brussels, Belgium; 3 Faculty of Medicine, University of Bern, Murtenstrasse 10, Bern, 3008 Switzerland; 4 Institute of Social and Preventive Medicine, University of Bern, Bern, Switzerland

**Keywords:** Low Back Pain, Musculoskeletal Pain, Switzerland, Risk Factors, Physical Activity, Exercise, Life Style, Cross-Sectional Studies

## Abstract

**Background:**

Low back pain (LBP) affects about 1.2 million individuals in Switzerland. Although regular leisure-time physical activity (PA) is considered a relevant factor in preventing LBP, evidence of its association with LBP is inconsistent. This study investigated the association between leisure-time PA and LBP, as well as the relationship with other factors.

**Methods:**

We analysed data from the cross-sectional 2022 Swiss Health Survey which covered adults aged 18 years and older living in private households in Switzerland. Self-reported LBP in the previous four weeks was grouped as no, a little or strong. Multinomial logistic regression models were used to analyse LBP in relationship to predictors and sociodemographic characteristics of respondents. We report crude and adjusted odds ratios (OR) with 95% confidence intervals (CI).

**Results:**

Complete data from 13,418 individuals (mean age: 51.01 ± 16.32 years, 53.7% female) was analysed. Any LBP in the previous four weeks was reported by 40% of men and 50% of women. Strong LBP was more prevalent among men who were physically inactive in leisure time (13%) and women (17%) than among individuals who were physically trained in leisure time (men: 4.3%, women: 5.8%). Leisure-time PA was associated with lower odds of experiencing strong LBP, with the strongest protective association observed in physically trained individuals (adjusted OR = 0.35, 95% CI = [0.26; 0.49]). Associations with a little LBP were weaker and largely not statistically significant, except in women (adjusted OR = 0.73, 95% CI = [0.55; 0.97]). In the sex-specific models, moderate sleeping disorders and mild to moderate depression were associated with both a little and strong LBP in both sexes. Strong social relationships reduced the odds of both levels of LBP in women, while alcohol consumption increased the odds of a little LBP and smoking increased the odds of strong LBP in men.

**Conclusion:**

Respondents with increased levels of leisure-time PA had lower odds of strong LBP. Lifestyle factors such as sleep quality, social relationships, alcohol consumption and mental health contributed to the association with LBP, with sex-specific differences. Promoting leisure-time PA and other lifestyle factors may help to reduce the burden of LBP in the Swiss population. Further, ideally, prospective cohort studies are needed to confirm these results.

**Trial registration:**

Swiss Federal Statistical Office consent (Ref. 240286).

**Supplementary Information:**

The online version contains supplementary material available at https://doi.org/10.1186/s12891-026-10099-2.

## Background

Low back pain (LBP) has a lifetime prevalence worldwide of up to 84% [1] and affected approximately 1.2 million individuals in Switzerland in 2023, making it one of the most prevalent non-communicable diseases in the country [2]. This condition resulted in 138,561 disability-adjusted life years (DALY) in 2023 [2]. The economic burden of LBP in Switzerland was substantial, with total societal costs estimated at €4.8 billion and €6.6 billion [3]. Of this amount, €2.6 billion was attributed to direct healthcare costs, while productivity losses accounted for €2.19 to €4.08 billion [3]. About 4.4% of the participants of the cost of illness study in Switzerland [3] reported absenteeism due to LBP, meaning they did not attend scheduled work within the previous four weeks due to LBP [3, 4]. In contrast, presenteeism, defined as attending work despite experiencing LBP, accounted for 27.7% of total productivity losses [3, 4]. In relation to Switzerland’s total healthcare expenditure of €33.6 billion in 2005, approximately 6.1% were related to the annual healthcare costs for LBP, which is comparable to the annual healthcare expenditures for dental treatments (6.2%). This expenditure for LBP relates to 1.6 to 2.3% of the country’s Gross Domestic Product [3]. Consequently, LBP can be considered a significant public health issue [5].

Current scientific evidence indicates that various risk factors contribute to the development of LBP [6]. Non-modifiable risk factors include a history of LBP and a height greater than 170 centimetres [6]. At present, it is unclear whether sex differences exist for the risk of LBP [6]. According to the 2022 Swiss Health Survey (SHS), which is based on a representative sample of the permanent resident population in Switzerland, 50% of women and 40% of men reported experiencing back pain problems within the previous four weeks [7]. This might indicate an increased risk of LBP for women as compared to men [7].

In contrast, modifiable risk factors include poor overall health, physical stress (including occupational physical demands), psychological stress [6], and high body mass index (BMI) [8]. Among these, especially moderate leisure-time physical activity (PA) emerged as a key lifestyle pillar in preventing acute or chronic LBP [9–12]. The relationship between leisure-time PA in general and LBP remains less clear [9, 13–18]. Some systematic reviews found no association between LBP and PA levels [16–18], while another systematic review suggested a lower prevalence of LBP among physically active individuals [9]. Additionally, some studies indicated an increased risk for LBP for those who were either insufficiently or excessively active during leisure time [14] or in general [13, 15], which may point towards a U-shaped relationship [13–15].

Therefore, the goal of the present study was to evaluate the association between leisure-time PA and LBP as well as the evaluation of other risk factors for LBP (in the four weeks before answering the survey) for adults living in Switzerland by using the SHS from 2022.

The present study aimed to evaluate the following two research questions:

- What is the association between leisure-time PA and LBP in adults living in Switzerland in 2022?
- What other risk factors are associated with LBP in adults living in Switzerland in 2022?

We hypothesised that moderate levels of leisure-time PA are associated with a lower risk of LBP. Additionally, we proposed that certain risk factors, such as psychological factors, contribute to an increased risk of developing LBP.

## Methods

This manuscript followed the “Strengthening the Reporting of Observational Studies in Epidemiology” (STROBE) statement for the reporting of cross-sectional studies [19].

### Study design, setting and participants

To investigate the stated research questions, we analysed the data from the SHS of the year 2022. The SHS is a cross-sectional survey that is conducted every five years since 1992 in Switzerland [7]. This survey aims to analyse and enhance the healthcare system and associated services in response to the current needs of the Swiss population [20]. It consists of a telephone interview and a written survey to evaluate the physical and psychological health status, disease prevalence, and lifestyle factors impacting health [7]. All individuals living in Switzerland, aged 15 years and older, who have their own household and are not involved in an asylum process are eligible to participate in the study [20]. A stratified, multistage random sampling design is employed to ensure representativeness across demographic and regional stratification per canton [20]. Records are weighted by the Swiss Federal Statistical Office to extrapolate the data to the total population in Swiss private households [20]. Further methodological details can be found elsewhere [20].

The analytical study sample was not predetermined but rather derived from the available SHS dataset. The SHS from 2022 involved data including missing values from 21,930 telephone interviews and 19,137 individuals in the written survey. Only complete cases of adults aged 18 years and older were considered for the present analysis. The Swiss Federal Statistical Office gave written consent to analyse and publish the anonymised data (Ref. 240286). No further ethical approval was needed to work with these data.

### Outcome variables

Several questions from the 2022 survey were relevant to answer the research question of this present study. The sociodemographic variables included sex, age categories, BMI, civil status, nationality, language region and educational level.

The primary outcome was a three-level factor on back pain or LBP which was addressed in one question of the telephone interviews. Individuals were asked to tell whether they had such a kind of pain in the previous four weeks. This question was related to the German pain questionnaire [21] and covered three levels: “No pain”, “A little pain” and “Severe pain”.

The primary independent variable was leisure-time PA. The index variable was calculated since 2002 according to the same principle and based on the individual questions from the telephone interviews of the SHS. It included five levels of leisure-time PA, ranging from being physically inactive to being physically trained in leisure time. No information on the duration of work-related physical activities was available, and occupational status could not be reliably linked to the level of occupational PA (e.g., academic job). Therefore, reliable occupational PA was not available in the SHS.

Other potential risk factors for LBP were included based on the pillars of lifestyle medicine [22]. In addition to PA, these included a healthy diet, restful sleep, social relationships, sedentary behaviour and abstinence from substances such as alcohol and nicotine [22].

Furthermore, the model by Hartvigsen et al. [23] suggested additional risk factors, such as psychological factors (e.g. depression, anxiety), social factors or societal factors (e.g. level of education) as well as comorbidities (e.g. diabetes). Biophysical (e.g. alteration in muscle size) and genetic factors were not evaluated in the SHS. A summary of all considered variables is attached in Additional file 1.

### Statistical methods

To evaluate the research questions, data from the SHS from 2022 were merged and grouped by the variable IDNO in the wide format. The evaluation was conducted using complete cases and survey weights of the telephone survey (variable: WGHT). The dependent variable for back pain stayed as a three level categorial variable for the analysis with “no pain” serving as the reference category for the analysis. The PA variable was the primary independent variable and was five-level Likert scale ranging from the reference value “inactive” to “trained”. Additionally, to evaluate the second research question variables for the lifestyle pillars and the model by Hartvigsen were included [22, 23].

Descriptive statistics included the reporting of the number of respondents, stratified by sex, as well as the percentages of the population in each group and outcome of interest. All categorial variables were reported as percentages and continuous variables as means with standard deviations (SD).

To enhance technical validity, prevalence values of LBP were compared with the descriptive data reported by the Federal Statistical Office [24]. This included overall prevalence values, as well as those stratified by sex, age category and the combination of sex and age category.

Multinomial logistic regression models were fitted using complete cases only to evaluate the research questions. Sociodemographic variables (i.e., indicator variables for 10-year age groups, BMI groups, civil status, language regions and educational levels) were defined as confounders as these might distort the true relationship of the variables of interest.

Models one and two were used to answer the first research question, while the models three and four evaluated potential other influencing factors. The first model included only unadjusted PA levels and LBP (i.e., without confounders), while the second model involved the inclusion of confounders (i.e., sociodemographic variables) and was conducted separately for men and women (Additional file 2). The third model additionally included the lifestyle pillars, considered separately for both sexes. The fourth model included in addition to the third model the factors from the model by Hartvigsen et al. [23]. A detailed description of the four model formulas can be found in Additional file 2.

The associations of the predictors with LBP were reported as crude odds ratios (OR) and adjusted OR, accompanied by corresponding 95% confidence intervals (CI). Analyses were conducted separately by sex for the models two to four. The reference groups were the lowest level for ordered variables (e.g. leisure-time physical activity: inactive). The 45- to 54 year age group was used as the reference group for the variable ‘age category’, as it most closely reflected the mean age of the study population (Additional file 1). P-values below 0.05 were considered statistically significant. We did not adjust p-values for multiple analyses.

RStudio (version 4.5.0) [25] was used for the analysis including the packages ‘tidyverse’ package (version 2.0.0) [26] for data manipulation, ‘survey’ package (version 4.4-2) [27] and the ‘srvyr’ package (version 1.3.0) [28] for developing the survey design and weighted statistics. The ‘svyVGAM’ package version 1.2 [29] was used to develop the model, the ‘parameters’ package (version 0.24.2) [30] to generate the model output and the ‘gtsummary’ package (version 2.2.0) [31] for data reporting.

## Results

### Survey respondents

**Fig. 1:**
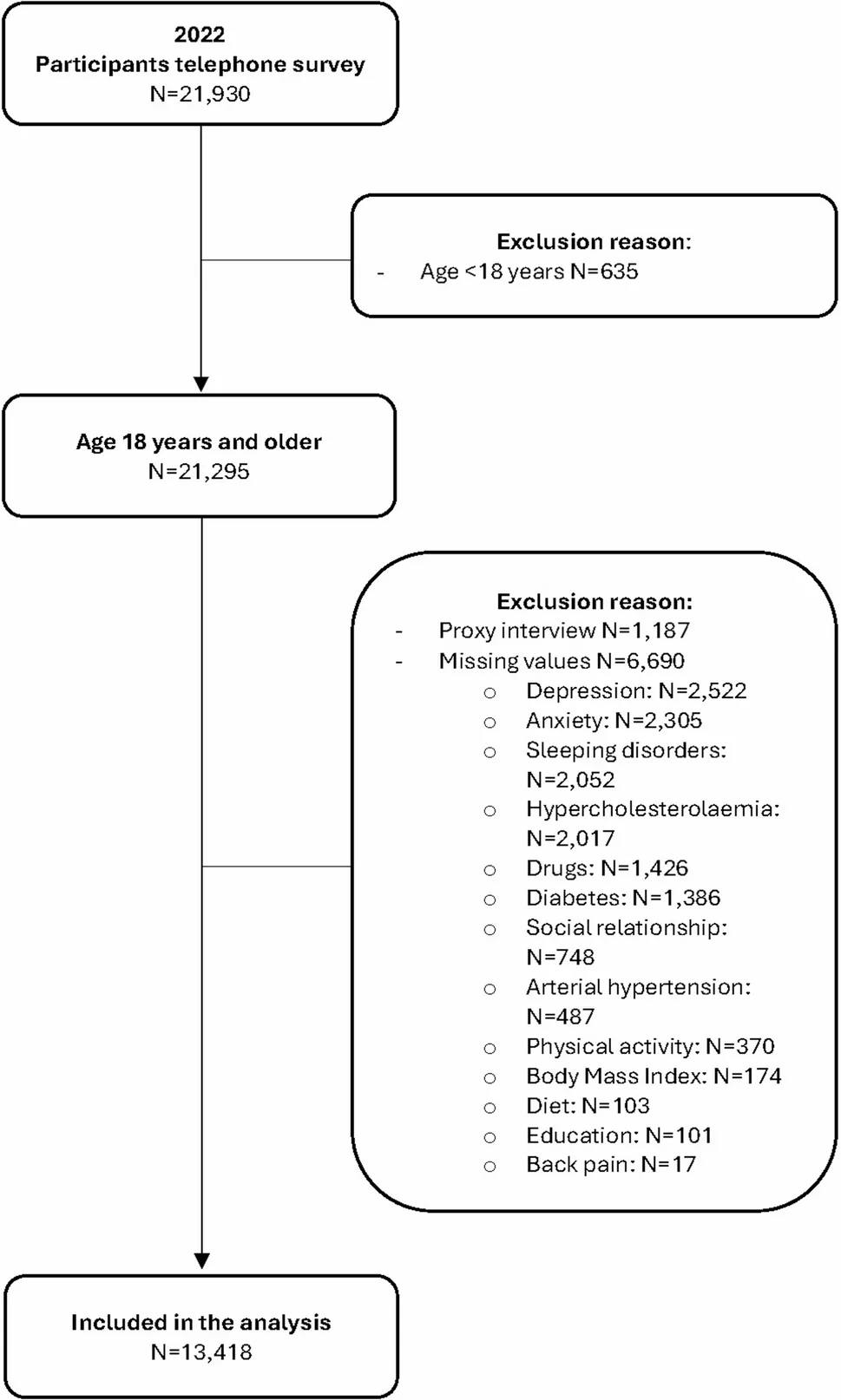
Flowchart of individuals included in the analysis. Legend: N = number of test subjects. The number of missing values exceeds the reported count, as values were missing in multiple variables

The telephone survey of the 2022 SHS was conducted with 21,930 individuals. Among these, 21,295 were aged 18 years and older. For the analysis, complete data were available for 13,418 individuals which were included in the final dataset (Fig. 1).

### Descriptive data

The 13,418 participants were on average 51.01 years old (SD: 16.32), with 53.7% (*N* = 7,207) of them being female. 55% (*N* = 7,397) of the participants reported no LBP, 37.7% (*N* = 5,064) experienced a little LBP, and 7.1% (*N* = 957) had strong LBP in the previous four weeks (Table 1). Among men (*N* = 6,211), 40% reported LBP of any intensity in the previous four weeks, compared to 50% of women (*N* = 7,207). A little LBP was reported by 34% of men and 41% of women, while strong LBP affected 5.3% of men and 8.7% of women (Additional file 3).

**Table 1:** Descriptive (weighted) statistics for low back pain, separated by sex

	Male			Female			Overall		
Characteristic	No *N* = 3,767	A little *N* = 2,109	Strong *N* = 335	No *N* = 3,630	A little *N* = 2,955	Strong *N* = 622	No *N* = 7,397	A little *N* = 5,064	Strong *N* = 957
Age									
18–24 years	69%	29%	1.7%	49%	45%	6.6%	59%	37%	4.2%
25–34 years	59%	38%	3.8%	49%	43%	8.0%	54%	40%	5.9%
35–44 years	62%	33%	4.7%	52%	39%	9.5%	57%	36%	7.2%
45–54 years	59%	35%	6.6%	50%	42%	8.1%	54%	38%	7.4%
55–64 years	60%	33%	7.0%	50%	40%	10%	55%	36%	8.7%
65–74 years	58%	35%	6.8%	54%	39%	7.7%	56%	37%	7.3%
75 + years	59%	36%	5.3%	49%	40%	11%	54%	38%	8.4%
BMI									
Normal weight	61%	35%	4.5%	53%	39%	7.6%	56%	38%	6.3%
Underweight	59%	33%	7.9%	52%	39%	8.3%	53%	38%	8.2%
Overweight	63%	32%	5.0%	46%	45%	9.5%	57%	36%	6.6%
Obesity	52%	39%	9.1%	44%	42%	14%	48%	40%	11%
Civil status									
Single	61%	35%	3.6%	51%	41%	7.0%	57%	38%	5.3%
Married	61%	33%	6.3%	51%	40%	8.8%	56%	37%	7.5%
Divorced	58%	35%	7.4%	47%	41%	12%	51%	39%	10%
Widowed	55%	40%	4.5%	50%	40%	11%	51%	40%	9.3%
Education									
Compulsory school	61%	35%	3.8%	44%	42%	14%	51%	39%	9.4%
Upper secondary education	57%	36%	7.0%	49%	41%	9.8%	53%	39%	8.5%
Tertiary level	63%	33%	4.1%	53%	40%	6.4%	59%	36%	5.2%
Nationality									
Swiss	61%	34%	5.0%	51%	41%	8.3%	56%	38%	6.7%
Foreigner	60%	33%	6.5%	48%	41%	10%	54%	37%	8.4%
Language regions of Switzerland									
German-speaking part	61%	34%	5.3%	51%	40%	8.4%	56%	37%	6.9%
French-speaking part	58%	36%	5.8%	47%	43%	9.8%	52%	40%	7.9%
Italian-speaking part	64%	31%	4.3%	52%	41%	7.6%	57%	36%	6.1%
Physical activity									
Inactive	55%	31%	13%	42%	41%	17%	48%	37%	15%
Partially active	59%	35%	6.1%	46%	44%	10%	52%	40%	8.4%
Irregularly active	58%	37%	4.7%	48%	43%	8.4%	53%	40%	6.6%
Regularly active	58%	37%	5.6%	51%	40%	9.3%	54%	38%	7.7%
Trained	65%	31%	4.3%	57%	37%	5.8%	61%	34%	5.0%

Weighted %; Sum per row

*Abbreviations*: *BMI* Body Mass Index, *N* Unweighted number of participants

Individuals with leisure-time physical inactivity were more likely to report LBP than those being physically trained (Table 1). While 48% of individuals who were physically inactive during their leisure time reported no LBP, this proportion increased to 61% among those who were physically trained individuals. This pattern was consistent across both sexes. Strong LBP was reported by 13% (men) and 17% (women) of the physically inactive individuals, compared to 4.3% (men) and 5.8% (women) among physically trained individuals.

Strong LBP prevalence was the highest among individuals aged 55 to 64 years (Table 1). Among young adults (18 to 24 years), 37% had a little LBP and 4.2% strong LBP, whereas individuals aged 55 to 64 years experienced a little LBP in 36% of the cases and strong LBP in 8.7% of the cases. Individuals with obesity were more likely to experience strong LBP (11%) compared to those with normal weight (6.3%) or overweight (6.6%).

Regarding civil status and educational, being divorced (5.3%) and having a compulsory school education (9.4%) showed the highest proportion of strong LBP. A table presenting the descriptive statistics for PA levels, stratified by sex, is provided in Additional file 4. Calculated LBP prevalence aligned with the reported values of the Swiss Federal Statistical Office.

### Association of leisure-time PA and LBP

**Fig. 2:**
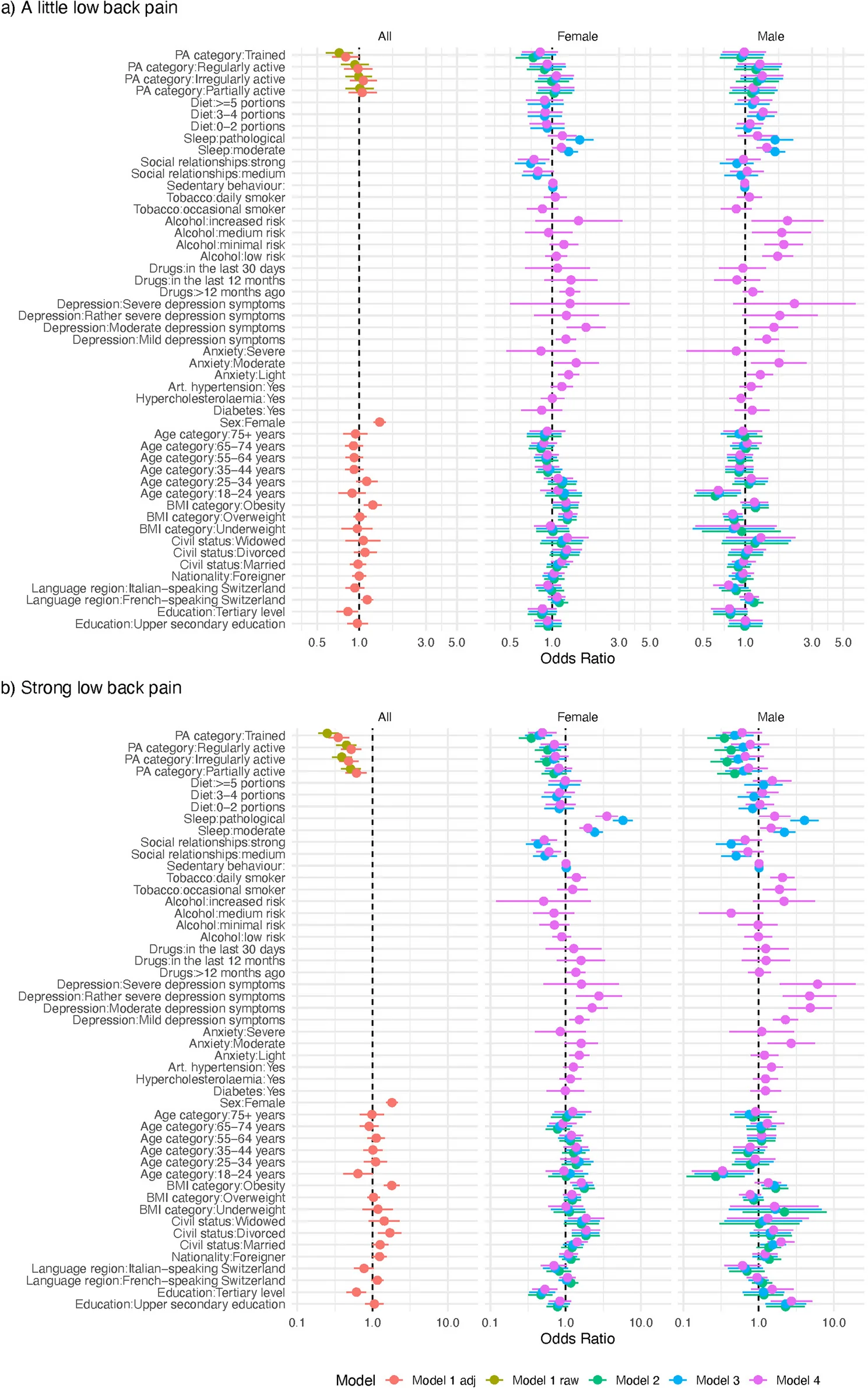
Forest plots of all models, separated by low back pain intensity; Abbreviations: BMI: Body Mass Index, PA: Physical Activity

For individuals reporting a little LBP, those classified as physically trained in their leisure time had statistically significant lower OR in the crude model one (crude OR: 0.72, 95% CI = [0.58; 0.90]) and in the model two for females (adjusted OR: 0.73, 95% CI = 0.55; 0.97]) compared to individuals who were physically inactive in leisure time (Additional file 5). This association was attenuated and no longer statistically significant, with adjusted ORs of 0.80 (95% CI = [0.64; 1.00]) in the adjusted model one and 0.93 (95% CI = [0.66; 1.33]) in model two for males. No other PA category showed statistically significant associations with a little LBP, when compared to individuals who were physically inactive in leisure time (Fig. 2).

In the unadjusted model one for strong LBP, all levels of PA were associated with lower ORs compared to inactive individuals, with the strongest association observed among individuals who were physically trained in leisure time (crude OR: 0.25, 95% CI = [0.19; 0.35], adjusted OR: 0.35, 95% CI = [0.26; 0.49]). The crude ORs decreased consistently with higher PA levels, except for regularly active individuals. The latter showed again higher crude OR (crude OR: 0.45, 95% CI = [0.33; 0.61]) and adjusted ORs (adjusted OR: 0.52, 95% CI = [0.38; 0.71]) compared to individuals who were irregularly active and physically trained in leisure time. In the sex-specific model two, these patterns were also apparent, with lower ORs for males compared to females (Fig. 2, Additional file 5). The strongest association remained for individuals who were physically trained in leisure time, with adjusted ORs of 0.35 (95% CI = [0.21; 0.58]) for males and 0.35 (95% CI = [0.24; 0.53]) for females. The only statistically non-significant adjusted OR values were found among female individuals who were partially active (adjusted OR: 0.70, 95% CI = [0.48; 1.03]).

Among the confounders in adjusted model one, female sex was associated with higher OR for LBP, with adjusted OR of 1.40 (95% CI = [1.27; 1.54]) for a little LBP and 1.81 (95% CI = [1.51; 2.16]) for strong LBP, when compared to males. The same applied for individuals with obesity with an adjusted OR of 1.25 (95% CI = [1.08; 1.45]) for a little LBP and 1.80 (95% CI = [1.41; 2.29]) for strong LBP compared to individuals with normal weight.

### Association of other risk factors and LBP

In male and female participants, moderate sleeping disorder was associated with an increased odds of a little LBP (adjusted OR 1.16 to 1.63 in model three and four). Similarly, mild to moderate depression symptoms (adjusted OR: 1.25 to 1.74) and light to moderate anxiety levels (adjusted OR: 1.28 to 1.74) increased the odds of a little LBP (Fig. 2).

In female participants, strong social relationships were associated with lower OR for a little LBP (adjusted OR values: 0.70 to 0.74), whereas in males, all levels of alcohol consumption increased the odds (adjusted OR values: 1.70 to 2.00). No other factors showed consistent statistically significant associations with a little LBP in models three and four.

Similar associations were observed for strong LBP. Moderate sleeping disorder and pathological sleep (adjusted OR: 1.46 to 5.78 in model 3 and 4), as well as mild to rather severe depression symptoms (adjusted OR: 1.52 to 4.84 in model 4) were associated with strong LBP in both sexes (Fig. 2). In females, medium to strong social relationships (adjusted OR: 0.52 to 0.60 in model 4) and tertiary education level (adjusted OR: 0.49 to 0.53 in model 3 and 4) were protective for strong LBP when compared to no social relationships. While light anxiety levels (adjusted OR: 1.52, 95% CI = [1.12; 2.06]) were associated with strong LBP in female participants, this was the case for moderate anxiety (adjusted OR: 2.70, 95% CI = [1.31; 5.55]) in male participants (Fig. 2). In male participants, occasional or daily smoking (adjusted OR: 1.89 to 2.07 in model 4) and upper secondary education (adjusted OR: 2.29 to 2.72 in model 3 and 4) were associated with strong LBP (Fig. 2). A detailed list of exact OR values is available in Additional file 5.

## Discussion

The present study examined the association between leisure-time PA and LBP among Swiss adults, and explored other factors associated with LBP. Based on data from the 2022 SHS, higher leisure-time PA was associated with lower odds of strong LBP, suggesting a potential protective effect. This association was weaker for a little LBP. Additionally, notable sex-specific differences were observed in the association between LBP and other lifestyle factors such as sleep quality, social relationships, alcohol consumption, and mental health.

The results on the association of leisure-time PA and LBP contrast previous systematic reviews that found no consistent association between the two variables [16–18]. The systematic reviews by Hendrick et al. [16], Sitthipornvorakul et al. [17] and Heneweer et al. [18] included both prospective cohort and cross-sectional studies on LBP, most of which relied on self-reported PA data. Only a small number of studies included objective measurements such as accelerometery [16–18]. The study conducted by Heneweer et al. [18] found inconsistent results for leisure-time PA, sports, and exercise. However, the study highlighted flexed, rotated and unusual positions of the lumbar spine, as well as heavy workloads and frequent lifting, as moderate to strong risk factors for LBP [18].

In contrast to these earlier findings, the present study showed a clear protective association between higher levels of leisure-time PA with strong LBP. One possible reason for this discrepancy may be due to the methodological difference. Unlike the aforementioned studies [16–18], the SHS data differentiated LBP by intensity, which may explain the clearer protective pattern observed for leisure-time PA. Risk factors such as flexed lumbar spine were not evaluated in the SHS thus of the present analysis.

Conversely to these findings, some studies have reported a U-shaped association between PA and LBP, whereby low and very high levels of PA were likely to increase the occurrence of LBP [13] or chronic LBP [14, 15]. Wider age categories of 20-year range were employed [14].

The absence of such a finding in the present study may be related to the categorical assessment of PA in the present study. The evaluation of PA levels in the present study may have a ceiling effect, since the “trained” level of the PA variable was defined according to the fulfilment of the World Health Organization (WHO) criteria. For example, highly trained individuals or athletes, who are also often affected by LBP [32] were in the same category as those just meeting the WHO criteria. One of the aforementioned studies which found a U-shaped association used the metabolic equivalent (MET) to measure weekly PA levels on a continuous scale [15].

Beyond PA, the present study adopted a more comprehensive perspective on the multifactorial nature of LBP by integrating additional lifestyle factors and factors presented by Hartvigsen et al. [23], such as sleep quality, social relationships and alcohol consumption. The current findings therefore extended this list of relevant risk factors, by applying them to the Swiss population, and thereby reinforcing the multifactorial nature of LBP [23]. In particular, the sex-specific patterns highlighted the need to consider this when preventing LBP. For example, social relationships emerged as an important protective factor for women, while this was not evident for men.

Despite the strengths of a large, nationally representative sample and multivariable adjustment, several limitations should be acknowledged. First, although the SHS applied random sampling, participation bias may still be present, as only 36% of contacted individuals participated [20]. Individuals with fewer or less severe symptoms may have been more likely to participate, potentially underestimating the true prevalence of LBP. In addition, self-reported measures may be subject to social desirability or reporting bias [33, 34], with participants reporting their health status in a more favourable way and thus overestimating PA and underreporting LBP. This emphasised the need for objective measurements such as e.g. accelerometers in PA.

Second, due to the cross-sectional design, the temporal order between PA and LBP could not be established, which is one of the Bradford Hill criteria used to evaluate causality [35]. Therefore, the present analyses identified associations rather than causal relationships. However, it remained unclear whether LBP reduced PA, or whether PA levels contributed to the development of LBP. The same applied for all other lifestyle factors, e.g. sleep. Multicollinearity between these lifestyle factors and musculoskeletal pain might also have affected the observed association, as these parameters are often intercorrelated [36].

Third, the present study only considered leisure-time PA, although occupational PA may have opposite health implications. The systematic review of Alzaharni et al. evaluated leisure-time PA to be a protective factor for LBP (OR: 0.85, 95% CI = [0.79; 0.93]) [9], whereas this association was not reported in the analysis of the general PA levels including both occupational and leisure-time PA (risk ratio (RR): 0.90, 95% CI = [0.92; 1.08]), when compared to low PA levels. Similarly, based on a Swedish (*N* = 5,798) [37] and Norwegian cohort study.

(*N* = 14,915) [38], more physically demanding and strenuous work and lower levels of leisure-time PA was associated with higher occurrence of LBP and potentially also chronic LBP (defined as LBP persistence of more than six months) [37, 38]. For example, occupational PA led to increased associations with chronic LBP with relative risk ratios of 1.30 (95% CI: 1.00-1.71) for women and 1.36 (95% CI: 1.17–1.59) for men [38]. It was also associated with long-term sickness absence, as evaluated in a Danish cohort study of 7,144 employees [12]. Distinguishing between PA contexts is therefore essential for future research.

Finally, as the SHS included community-dwelling adults aged 18 and older in Switzerland, the findings may not be applied to children, adolescents, individuals in institutional settings (e.g. hospitals), or those with severe disabilities.

Future prospective cohort studies should further evaluate the directionality of the association between LBP and PA and consider psychosocial and lifestyle, and sociodemographic factors. Moreover, distinguishing between leisure-time and occupational PA could help to better clarify the effects of different contexts of PA on LBP. Incorporating objective measures, such as accelerometers could improve measurement accuracy and reduce the reliance on self-reported data. Given the variability of LBP severity, a more continuous assessment of pain levels would also enhance the understanding of the association.

## Conclusion

This study evaluated the association between leisure-time PA with LBP, as well as other potential factors, in a Swiss cohort using data from the 2022 SHS. The results indicated an association between leisure-time PA and LBP; particularly in strong LBP cases, with higher PA values being protective. Aside from PA, other lifestyle factors encompassing sleep, mental health, social relationships, and substance use (alcohol, tobacco) were also found to influence the occurrence of LBP, with notable sex-specific variations. Further prospective cohort studies are needed to investigate these associations further. Differentiating between different PA contexts (e.g. leisure time and occupational PA) and considering objective measurement tools to measure PA could also enhance accuracy.

## Supplementary Information

Supplementary Material 1.

Supplementary Material 2.

Supplementary Material 3.

Supplementary Material 4.

Supplementary Material 5.

## Data Availability

This is a secondary data analysis. We have an agreement with the Swiss Federal Statistical Office to analyse the anonymous data (Ref. 240286). The raw data are not available. However, the underlying data analysis is available at: https://rpubs.com/sua1/lbp-pa-shs2022.

## Abbreviations

BMI: Body mass index
CI: Confidence interval
DALY: Disability-adjusted life years
LBP: Low back pain
MET: Metabolic equivalent
OR: Odds ratio
PA: Physical activity
RR: Risk ratio
SD: Standard deviation
SHS: Swiss Health Survey
STROBE: Strengthening the Reporting of Observational Studies in Epidemiology

## References

[1] Airaksinen O, Brox JI, Cedraschi C, Hildebrandt J, Klaber-Moffett J, Kovacs F, et al. Chapter 4 European guidelines for the management of chronic nonspecific low back pain. Eur Spine J. 2006;15(Suppl 2):s192–300. 10.1007/s00586-006-1072-1 PMC3454542 16550448

[2] Institute for Health Metrics and Evaluation. Results from the 2023 Global Burden of Disease (GBD) study. 2025. GBD Results Tool. Available from: https://vizhub.healthdata.org/gbd-results. Cited 20 Oct 2025.

[3] Wieser S, Horisberger B, Schmidhauser S, Eisenring C, Brügger U, Ruckstuhl A, et al. Cost of low back pain in Switzerland in 2005. Eur J Health Econ. 2011;12(5):455–67. 10.1007/s10198-010-0258-y PMC3160551 20526649

[4] Lohaus D, Habermann W, Presenteeism. A review and research directions. Hum Resource Manage Rev. 2019;29(1):43–58.

[5] Balagué F, Mannion AF, Pellisé F, Cedraschi C. Non-specific low back pain. Lancet. 2012;379(9814):482–91. 10.1016/S0140-6736(11)60610-7 21982256

[6] Parreira P, Maher CG, Steffens D, Hancock MJ, Ferreira ML. Risk factors for low back pain and sciatica: an umbrella review. Spine J. 2018;18(9):1715–21. 10.1016/j.spinee.2018.05.018 29792997

[7] Bundesamt für Statistik. Schweizerische Gesundheitsbefragung - Körperliche Beschwerden (1 von 2) – 1992. 1997, 2002, 2007, 2012, 2017, 2022. Available from: https://www.bfs.admin.ch/asset/de/28845228. Cited 22 Nov 2024.

[8] Roberts KE, Ferreira ML, Beckenkamp PR, Nicholson S, March L, Ferreira PH. Global Trends in Risk Factors for Low Back Pain: An Analysis of the Global Burden of Disease Study Data From 1990 to 2021. Arthritis Care Res. 2025;77(7):837–47. 10.1002/acr.25520 PMC12209496 40051120

[9] Alzahrani H, Mackey M, Stamatakis E, Zadro JR, Shirley D. The association between physical activity and low back pain: a systematic review and meta-analysis of observational studies. Sci Rep. 2019;9(1):8244. 10.1038/s41598-019-44664-8 PMC6547713 31160632

[10] Shiri R, Falah-Hassani K. Does leisure time physical activity protect against low back pain? Systematic review and meta-analysis of 36 prospective cohort studies. Br J Sports Med. 2017;51(19):1410–8. 10.1136/bjsports-2016-097352 28615218

[11] Steffens D, Maher CG, Pereira LSM, Stevens ML, Oliveira VC, Chapple M, et al. Prevention of Low Back Pain: A Systematic Review and Meta-analysis. JAMA Intern Med. 2016;176(2):199–208. 10.1001/jamainternmed.2015.7431 26752509

[12] Holtermann A, Hansen JV, Burr H, Søgaard K, Sjøgaard G. The health paradox of occupational and leisure-time physical activity. Br J Sports Med. 2012;46(4):291–5. 10.1136/bjsm.2010.079582 21459873

[13] Heneweer H, Vanhees L, Picavet HSJ. Physical activity and low back pain: a U-shaped relation? Pain. 2009;143(1–2):21–5. 10.1016/j.pain.2008.12.033 19217208

[14] Heuch I, Heuch I, Hagen K, Zwart JA. Is there a U-shaped relationship between physical activity in leisure time and risk of chronic low back pain? A follow-up in the HUNT Study. BMC Public Health. 2016;16:306. 10.1186/s12889-016-2970-8 PMC4827170 27068452

[15] Ziegelmayer S, Häntze H, Mertens C, Busch F, Lemke T, Kather JN et al. Intermuscular adipose tissue and lean muscle mass assessed with MRI in people with chronic back pain in Germany: a retrospective observational study. The Lancet Regional Health – Europe. 2025;54. Available from: https://www.thelancet.com/journals/lanepe/article/PIIS2666-7762(25)00115-2/fulltext. Cited 4 Jul 2025. 10.1016/j.lanepe.2025.101323 PMC12141060 40487774

[16] Hendrick P, Milosavljevic S, Hale L, Hurley DA, McDonough S, Ryan B, et al. The relationship between physical activity and low back pain outcomes: a systematic review of observational studies. Eur Spine J. 2011;20(3):464–74. 10.1007/s00586-010-1616-2 PMC3048226 21053026

[17] Sitthipornvorakul E, Janwantanakul P, Purepong N, Pensri P, van der Beek AJ. The association between physical activity and neck and low back pain: a systematic review. Eur Spine J. 2011;20(5):677–89. 10.1007/s00586-010-1630-4 PMC3082686 21113635

[18] Heneweer H, Staes F, Aufdemkampe G, van Rijn M, Vanhees L. Physical activity and low back pain: a systematic review of recent literature. Eur Spine J. 2011;20(6):826–45. 10.1007/s00586-010-1680-7 PMC3099170 21221663

[19] von Elm E, Altman DG, Egger M, Pocock SJ, Gøtzsche PC, Vandenbroucke JP, et al. The Strengthening the Reporting of Observational Studies in Epidemiology (STROBE) statement: guidelines for reporting observational studies. J Clin Epidemiol. 2008;61(4):344–9. 10.1016/j.jclinepi.2007.11.008 18313558

[20] Bundesamt für Statistik. Die Schweizerische Gesundheitsbefragung 2022 in Kürze - Konzept, Methode, Durchführung | Publikation. 2022. Die Schweizerische Gesundheitsbefragung 2022 in Kürze - Konzept, Methode, Durchführung | Publikation. Available from: https://www.bfs.admin.ch/asset/de/31646339. Cited 7 Feb 2025.

[21] Deutsche Schmerzgesellschaft e.V. Deutscher Schmerzfragebogen. 2024. Deutscher Schmerzfragebogen. Available from: https://www.schmerzgesellschaft.de/schmerzfragebogen. Cited 30 Dec 2024.

[22] Rippe JM. Lifestyle Medicine: The Health Promoting Power of Daily Habits and Practices. Am J Lifestyle Med. 2018;12(6):499–512. 10.1177/1559827618785554 PMC6367881 30783405

[23] Hartvigsen J, Hancock MJ, Kongsted A, Louw Q, Ferreira ML, Genevay S, et al. What low back pain is and why we need to pay attention. Lancet. 2018;391(10137):2356–67. 10.1016/S0140-6736(18)30480-X 29573870

[24] Bundesamt für Statistik. Ergebnisse und Publikationen.Ergebnisse der Schweizerischen Gesundheitsbefragung. Ergebnisse und Publikationen. Available from: https://www.bfs.admin.ch/bfs/de/home/statistiken/gesundheit/erhebungen/sgb/ergebnisse-publikationen.html. Cited 17 Jan 2025.

[25] R Core Team. R: A language and environment for statistical computing. Vienna, Austria. 2025. Available from: https://www.r-project.org/. Cited 30 Apr 2025.

[26] Wickham H, Averick M, Bryan J, Chang W, McGowan LD, François R, et al. Welcome to the Tidyverse. J Open Source Softw. 2019;4(43):1686.

[27] Lumley T. Wiley.com. 2010. Complex Surveys: A Guide to Analysis Using R. Available from: https://www.wiley.com/en-us/Complex+Surveys%3A+A+Guide+to+Analysis+Using+R-p-9781118210932. Cited 30 Apr 2025.

[28] Ellis GF, Lumley T, Żółtak T, Schneider B, Krivitsky PN. srvyr: ’dplyr’-Like Syntax for Summary Statistics of Survey Data. 2024. Available from: https://cran.r-project.org/web/packages/srvyr/index.html. Cited 1 May 2025.

[29] Lumley T, svyVGAM. Design-Based Inference in Vector Generalised Linear Models. 2023. Available from: https://cran.r-project.org/web/packages/svyVGAM/index.html. Cited 30 Apr 2025.

[30] Lüdecke D, Ben Shachar M, Patil I, Makowski D. Extracting, Computing and Exploring the Parameters of Statistical Models using R. J Open Source Softw. 2020;5:2445.

[31] Sjoberg DD, Whiting K, Curry M, Lavery JA, Larmarange J. Reproducible Summary Tables with the gtsummary Package. R J. 2021;13(1):570.

[32] Schmidt CP, Zwingenberger S, Walther A, Reuter U, Kasten P, Seifert J, et al. Prevalence of low back pain in adolescent athletes - an epidemiological investigation. Int J Sports Med. 2014;35(8):684–9. 10.1055/s-0033-1358731 24424960

[33] Zhu OY, Greene D, Dolnicar S. Should the risk of social desirability bias in survey studies be assessed at the level of each pro-environmental behaviour? Tour Manag. 2024;104:104933.

[34] Krumpal I. Determinants of social desirability bias in sensitive surveys: a literature review. Qual Quant. 2013;47(4):2025–47.

[35] Shimonovich M, Pearce A, Thomson H, Keyes K, Katikireddi SV. Assessing causality in epidemiology: revisiting Bradford Hill to incorporate developments in causal thinking. Eur J Epidemiol. 2021;36(9):873–87. 10.1007/s10654-020-00703-7 PMC8206235 33324996

[36] Kyriazos T, Poga M. Dealing with Multicollinearity in Factor Analysis: The Problem, Detections, and Solutions. OJS. 2023;13(03):404–24.

[37] van Björck-van Dijken CB, Wiklund AF, Hildingsson C. Low back pain, lifestyle factors and physical activity: a population based-study. J Rehabil Med. 2008;40(10):864–9. 10.2340/16501977-0273 19242625

[38] Heuch I, Heuch I, Hagen K, Zwart JA. Physical activity level at work and risk of chronic low back pain: A follow-up in the Nord-Trøndelag Health Study. PLoS ONE. 2017;12(4):e0175086. 10.1371/journal.pone.0175086 PMC5386240 28394896

