# Supplementary material for: Leisure-time physical activity and low back pain in Switzerland: results from the 2022 cross-sectional Swiss Health Survey
Source: BMC Musculoskelet Disord. 2026 Jun 30;27:808. doi: 10.1186/s12891-026-10099-2 (PMC13621532; doi:10.1186/s12891-026-10099-2)
Supplement: Supplementary file 1 [file 12891_2026_10099_MOESM1_ESM.pdf]

## Additional file 5. Model outputs

***Model 1: Unadjusted and adjusted model for association of low back pain and physical activity.***

| LBP | Variable | Parameter | crude OR | crude 95% CI | adjusted OR | adjusted 95% CI |
|---|---|---|---|---|---|---|
| A little LBP | Physical activity level | Partially active | 1.01 | [0.80; 1.27] | 1.05 | [0.84; 1.33] |
| | Physical activity level | Irregularly active | 0.99 | [0.80; 1.23] | 1.07 | [0.86; 1.33] |
| | Physical activity level | Regularly active | 0.93 | [0.74; 1.17] | 0.98 | [0.78; 1.24] |
| | Physical activity level | Trained | 0.72 | [0.58; 0.90] | 0.80 | [0.64; 1.00] |
| | Sex | Female | | | 1.40 | [1.27; 1.54] |
| | Age category | 18-24 years | | | 0.89 | [0.71; 1.11] |
| | Age category | 25-34 years | | | 1.13 | [0.95; 1.35] |
| | Age category | 35-44 years | | | 0.92 | [0.79; 1.07] |
| | Age category | 55-64 years | | | 0.92 | [0.80; 1.06] |
| | Age category | 65-74 years | | | 0.91 | [0.79; 1.06] |
| | Age category | 75+ years | | | 0.94 | [0.77; 1.14] |
| | BMI category | Underweight | | | 0.97 | [0.75; 1.24] |
| | BMI category | Overweight | | | 1.01 | [0.91; 1.13] |
| | BMI category | Obesity | | | 1.25 | [1.08; 1.45] |
| | Civil status | Married | | | 0.98 | [0.86; 1.12] |
| | Civil status | Divorced | | | 1.10 | [0.92; 1.33] |
| | Civil status | Widowed | | | 1.07 | [0.80; 1.42] |
| | Nationality | Foreigner | | | 1.00 | [0.89; 1.12] |
| | Language region | French-speaking Switzerland | | | 1.14 | [1.03; 1.26] |
| | Language region | Italian-speaking Switzerland | | | 0.93 | [0.80; 1.09] |

| LBP | Variable | Parameter | crude OR | crude 95% CI | adjusted OR | adjusted 95% CI |
|---|---|---|---|---|---|---|
| | Education | Upper secondary education | | | 0.97 | [0.82; 1.15] |
| | Education | Tertiary level | | | 0.83 | [0.69; 0.98] |
| Strong LBP | Physical activity level | Partially active | 0.51 | [0.38; 0.70] | 0.61 | [0.44; 0.83] |
| | Physical activity level | Irregularly active | 0.39 | [0.29; 0.53] | 0.48 | [0.36; 0.65] |
| | Physical activity level | Regularly active | 0.45 | [0.33; 0.61] | 0.52 | [0.38; 0.71] |
| | Physical activity level | Trained | 0.25 | [0.19; 0.35] | 0.35 | [0.26; 0.49] |
| | Sex | Female | | | 1.81 | [1.51; 2.16] |
| | Age category | 18-24 years | | | 0.64 | [0.41; 1.01] |
| | Age category | 25-34 years | | | 1.10 | [0.77; 1.55] |
| | Age category | 35-44 years | | | 1.01 | [0.76; 1.35] |
| | Age category | 55-64 years | | | 1.12 | [0.86; 1.46] |
| | Age category | 65-74 years | | | 0.90 | [0.68; 1.20] |
| | Age category | 75+ years | | | 0.98 | [0.68; 1.42] |
| | BMI category | Underweight | | | 1.18 | [0.74; 1.87] |
| | BMI category | Overweight | | | 1.03 | [0.85; 1.26] |
| | BMI category | Obesity | | | 1.80 | [1.41; 2.29] |
| | Civil status | Married | | | 1.26 | [0.99; 1.62] |
| | Civil status | Divorced | | | 1.70 | [1.19; 2.41] |
| | Civil status | Widowed | | | 1.43 | [0.89; 2.28] |
| | Nationality | Foreigner | | | 1.24 | [0.99; 1.54] |
| | Language region | French-speaking Switzerland | | | 1.16 | [0.96; 1.40] |
| | Language region | Italian-speaking Switzerland | | | 0.77 | [0.57; 1.04] |

| LBP | Variable | Parameter | crude OR | crude 95% CI | adjusted OR | adjusted 95% CI |
|---|---|---|---|---|---|---|
| | Education | Upper secondary education | | | 1.06 | [0.80; 1.40] |
| | Education | Tertiary level | | | 0.61 | [0.45; 0.82] |

***Model 2: Adjusted model for association of low back pain and physical activity, separated by sex.***

| LBP | Variable | Parameter | adjusted OR (male) | 95% CI (male) | adjusted OR (female) | 95% CI (female) |
|---|---|---|---|---|---|---|
| A little LBP | Physical activity level | Partially active | 1.11 | [0.77; 1.61] | 1.03 | [0.77; 1.38] |
| | Physical activity level | Irregularly active | 1.22 | [0.86; 1.74] | 0.99 | [0.75; 1.31] |
| | Physical activity level | Regularly active | 1.19 | [0.83; 1.73] | 0.88 | [0.66; 1.17] |
| | Physical activity level | Trained | 0.93 | [0.66; 1.33] | 0.73 | [0.55; 0.97] |
| | Age category | 18-24 years | 0.61 | [0.43; 0.87] | 1.20 | [0.89; 1.62] |
| | Age category | 25-34 years | 1.06 | [0.81; 1.38] | 1.17 | [0.93; 1.48] |
| | Age category | 35-44 years | 0.90 | [0.72; 1.13] | 0.93 | [0.77; 1.14] |
| | Age category | 55-64 years | 0.92 | [0.75; 1.13] | 0.91 | [0.76; 1.1] |
| | Age category | 65-74 years | 1.01 | [0.81; 1.25] | 0.83 | [0.68; 1.01] |
| | Age category | 75+ years | 0.99 | [0.74; 1.32] | 0.88 | [0.66; 1.16] |
| | BMI category | Underweight | 0.94 | [0.49; 1.79] | 1.01 | [0.77; 1.32] |
| | BMI category | Overweight | 0.83 | [0.71; 0.97] | 1.28 | [1.1; 1.49] |
| | BMI category | Obesity | 1.18 | [0.95; 1.46] | 1.25 | [1.01; 1.53] |
| | Civil status | Married | 0.89 | [0.74; 1.08] | 1.08 | [0.91; 1.28] |
| | Civil status | Divorced | 1.00 | [0.75; 1.33] | 1.22 | [0.96; 1.56] |
| | Civil status | Widowed | 1.17 | [0.68; 2.03] | 1.16 | [0.82; 1.62] |
| | Nationality | Foreigner | 0.95 | [0.8; 1.14] | 1.03 | [0.88; 1.22] |
| | Language region | French-speaking Switzerland | 1.15 | [0.99; 1.34] | 1.12 | [0.98; 1.29] |
| | Language region | Italian-speaking Switzerland | 0.86 | [0.68; 1.09] | 0.99 | [0.8; 1.21] |
| | Education | Upper secondary education | 0.99 | [0.75; 1.31] | 0.93 | [0.75; 1.16] |
| | Education | Tertiary level | 0.78 | [0.59; 1.03] | 0.84 | [0.67; 1.06] |

| LBP | Variable | Parameter | adjusted OR (male) | 95% CI (male) | adjusted OR (female) | 95% CI (female) |
|---|---|---|---|---|---|---|
| Strong LBP | Physical activity level | Partially active | 0.48 | [0.28; 0.81] | 0.70 | [0.48; 1.03] |
| | Physical activity level | Irregularly active | 0.38 | [0.23; 0.61] | 0.56 | [0.39; 0.82] |
| | Physical activity level | Regularly active | 0.43 | [0.26; 0.71] | 0.58 | [0.39; 0.86] |
| | Physical activity level | Trained | 0.35 | [0.21; 0.58] | 0.35 | [0.24; 0.53] |
| | Age category | 18-24 years | 0.27 | [0.11; 0.65] | 1.02 | [0.59; 1.75] |
| | Age category | 25-34 years | 0.78 | [0.44; 1.38] | 1.38 | [0.89; 2.15] |
| | Age category | 35-44 years | 0.72 | [0.44; 1.17] | 1.28 | [0.9; 1.83] |
| | Age category | 55-64 years | 1.09 | [0.72; 1.67] | 1.16 | [0.83; 1.61] |
| | Age category | 65-74 years | 1.08 | [0.69; 1.69] | 0.78 | [0.54; 1.13] |
| | Age category | 75+ years | 0.84 | [0.48; 1.49] | 1.03 | [0.64; 1.66] |
| | BMI category | Underweight | 2.20 | [0.61; 7.96] | 1.12 | [0.69; 1.83] |
| | BMI category | Overweight | 0.86 | [0.63; 1.17] | 1.23 | [0.95; 1.6] |
| | BMI category | Obesity | 1.68 | [1.14; 2.46] | 1.77 | [1.29; 2.43] |
| | Civil status | Married | 1.36 | [0.93; 1.99] | 1.22 | [0.88; 1.68] |
| | Civil status | Divorced | 1.45 | [0.77; 2.74] | 1.86 | [1.22; 2.84] |
| | Civil status | Widowed | 1.02 | [0.3; 3.47] | 1.62 | [0.95; 2.77] |
| | Nationality | Foreigner | 1.38 | [0.96; 1.98] | 1.18 | [0.9; 1.54] |
| | Language region | French-speaking Switzerland | 1.12 | [0.82; 1.51] | 1.17 | [0.93; 1.48] |
| | Language region | Italian-speaking Switzerland | 0.70 | [0.41; 1.19] | 0.82 | [0.56; 1.19] |
| | Education | Upper secondary education | 2.27 | [1.26; 4.08] | 0.78 | [0.56; 1.08] |
| | Education | Tertiary level | 1.17 | [0.64; 2.13] | 0.47 | [0.32; 0.67] |

***Model 3: Adjusted model for association of low back pain, physical activity and lifestyle factors, separated by sex.***

| LBP | Variable | Parameter | Adjusted OR (male) | 95% CI (male) | Adjusted OR (female) | 95% CI (female) |
|---|---|---|---|---|---|---|
| A little LBP | Physical activity level | Partially active | 1.17 | [0.81; 1.70] | 1.06 | [0.79; 1.43] |
| | Physical activity level | Irregularly active | 1.30 | [0.92; 1.85] | 1.05 | [0.79; 1.40] |
| | Physical activity level | Regularly active | 1.24 | [0.86; 1.80] | 0.93 | [0.69; 1.25] |
| | Physical activity level | Trained | 0.96 | [0.67; 1.36] | 0.79 | [0.60; 1.06] |
| | Diet | 0-2 portions | 1.04 | [0.85; 1.29] | 0.92 | [0.70; 1.22] |
| | Diet | 3-4 portions | 1.29 | [1.03; 1.61] | 0.88 | [0.67; 1.17] |
| | Diet | ≥5 portions | 1.12 | [0.84; 1.49] | 0.90 | [0.67; 1.20] |
| | Sleep | moderate | 1.63 | [1.38; 1.92] | 1.31 | [1.14; 1.52] |
| | Sleep | pathological | 1.63 | [1.21; 2.20] | 1.57 | [1.26; 1.96] |
| | Social relationships | medium | 0.93 | [0.71; 1.23] | 0.78 | [0.61; 0.99] |
| | Social relationships | strong | 0.87 | [0.66; 1.14] | 0.70 | [0.54; 0.89] |
| | Sedentary behaviour | | 0.99 | [0.97; 1.02] | 1.01 | [0.99; 1.04] |
| | Age category | 18-24 years | 0.65 | [0.45; 0.93] | 1.22 | [0.91; 1.63] |
| | Age category | 25-34 years | 1.10 | [0.84; 1.44] | 1.19 | [0.94; 1.50] |
| | Age category | 35-44 years | 0.91 | [0.72; 1.14] | 0.96 | [0.79; 1.18] |
| | Age category | 55-64 years | 0.91 | [0.74; 1.12] | 0.90 | [0.74; 1.08] |
| | Age category | 65-74 years | 0.97 | [0.78; 1.21] | 0.84 | [0.69; 1.03] |
| | Age category | 75+ years | 0.90 | [0.67; 1.21] | 0.88 | [0.66; 1.16] |
| | BMI category | Underweight | 0.82 | [0.42; 1.61] | 1.00 | [0.77; 1.31] |
| | BMI category | Overweight | 0.82 | [0.70; 0.96] | 1.28 | [1.10; 1.49] |
| | BMI category | Obesity | 1.18 | [0.95; 1.46] | 1.25 | [1.01; 1.53] |

| LBP | Variable | Parameter | Adjusted OR (male) | 95% CI (male) | Adjusted OR (female) | 95% CI (female) |
|---|---|---|---|---|---|---|
| | Civil status | Married | 0.90 | [0.75; 1.10] | 1.10 | [0.92; 1.32] |
| | Civil status | Divorced | 1.01 | [0.76; 1.34] | 1.24 | [0.97; 1.58] |
| | Civil status | Widowed | 1.21 | [0.69; 2.12] | 1.18 | [0.84; 1.66] |
| | Nationality | Foreigner | 0.91 | [0.76; 1.09] | 1.00 | [0.85; 1.17] |
| | Language region | French-speaking Switzerland | 1.07 | [0.92; 1.26] | 1.07 | [0.93; 1.23] |
| | Language region | Italian-speaking Switzerland | 0.83 | [0.65; 1.05] | 0.95 | [0.77; 1.17] |
| | Education | Upper secondary education | 1.01 | [0.77; 1.33] | 0.94 | [0.76; 1.17] |
| | Education | Tertiary level | 0.79 | [0.59; 1.04] | 0.86 | [0.68; 1.08] |
| Strong LBP | Physical activity level | Partially active | 0.63 | [0.36; 1.10] | 0.79 | [0.53; 1.18] |
| | Physical activity level | Irregularly active | 0.53 | [0.31; 0.90] | 0.69 | [0.46; 1.01] |
| | Physical activity level | Regularly active | 0.62 | [0.35; 1.07] | 0.68 | [0.45; 1.02] |
| | Physical activity level | Trained | 0.48 | [0.27; 0.85] | 0.44 | [0.29; 0.66] |
| | Diet | 0-2 portions | 0.83 | [0.54; 1.28] | 0.82 | [0.52; 1.29] |
| | Diet | 3-4 portions | 0.86 | [0.53; 1.39] | 0.76 | [0.48; 1.19] |
| | Diet | ≥5 portions | 1.16 | [0.65; 2.06] | 0.95 | [0.59; 1.55] |
| | Sleep | moderate | 2.19 | [1.58; 3.05] | 2.43 | [1.91; 3.11] |
| | Sleep | pathological | 4.06 | [2.64; 6.24] | 5.78 | [4.27; 7.82] |
| | Social relationships | medium | 0.50 | [0.32; 0.79] | 0.53 | [0.37; 0.76] |
| | Social relationships | strong | 0.43 | [0.27; 0.69] | 0.43 | [0.30; 0.62] |
| | Sedentary behaviour | | 1.01 | [0.96; 1.06] | 1.02 | [0.97; 1.06] |
| | Age category | 18-24 years | 0.34 | [0.14; 0.84] | 1.15 | [0.67; 1.99] |
| | Age category | 25-34 years | 0.87 | [0.48; 1.57] | 1.51 | [0.98; 2.34] |

| LBP | Variable | Parameter | Adjusted OR (male) | 95% CI (male) | Adjusted OR (female) | 95% CI (female) |
|---|---|---|---|---|---|---|
| | Age category | 35-44 years | 0.75 | [0.46; 1.22] | 1.44 | [1.00; 2.08] |
| | Age category | 55-64 years | 1.09 | [0.70; 1.68] | 1.12 | [0.80; 1.58] |
| | Age category | 65-74 years | 1.07 | [0.68; 1.70] | 0.83 | [0.57; 1.22] |
| | Age category | 75+ years | 0.75 | [0.42; 1.37] | 1.11 | [0.67; 1.84] |
| | BMI category | Underweight | 1.66 | [0.40; 6.89] | 1.08 | [0.64; 1.81] |
| | BMI category | Overweight | 0.84 | [0.61; 1.15] | 1.23 | [0.95; 1.60] |
| | BMI category | Obesity | 1.60 | [1.09; 2.36] | 1.71 | [1.23; 2.37] |
| | Civil status | Married | 1.54 | [1.04; 2.29] | 1.26 | [0.92; 1.74] |
| | Civil status | Divorced | 1.42 | [0.76; 2.66] | 1.84 | [1.21; 2.81] |
| | Civil status | Widowed | 1.15 | [0.35; 3.78] | 1.65 | [0.96; 2.84] |
| | Nationality | Foreigner | 1.21 | [0.83; 1.78] | 1.08 | [0.82; 1.42] |
| | Language region | French-speaking Switzerland | 1.00 | [0.73; 1.36] | 1.03 | [0.81; 1.31] |
| | Language region | Italian-speaking Switzerland | 0.66 | [0.39; 1.13] | 0.73 | [0.50; 1.07] |
| | Education | Upper secondary education | 2.29 | [1.23; 4.26] | 0.81 | [0.58; 1.14] |
| | Education | Tertiary level | 1.15 | [0.61; 2.18] | 0.49 | [0.33; 0.72] |

***Model 4: Adjusted model for association of low back pain, physical activity, lifestyle factors and factors of the Hartvigsen model, separated by sex.***

| LBP | Variable | Parameter | Adjusted OR (male) | 95% CI (male) | Adjusted OR (female) | 95% CI (female) |
|---|---|---|---|---|---|---|
| A little LBP | Physical activity level | Partially active | 1.14 | [0.78; 1.65] | 1.06 | [0.79; 1.43] |
| | Physical activity level | Irregularly active | 1.32 | [0.93; 1.87] | 1.07 | [0.80; 1.42] |
| | Physical activity level | Regularly active | 1.27 | [0.88; 1.83] | 0.92 | [0.69; 1.24] |
| | Physical activity level | Trained | 0.98 | [0.69; 1.40] | 0.82 | [0.61; 1.09] |
| | Diet | 0-2 portions | 1.08 | [0.87; 1.34] | 0.91 | [0.69; 1.22] |
| | Diet | 3-4 portions | 1.34 | [1.06; 1.68] | 0.89 | [0.67; 1.18] |
| | Diet | ≥5 portions | 1.17 | [0.88; 1.55] | 0.88 | [0.65; 1.19] |
| | Sleep | moderate | 1.42 | [1.20; 1.68] | 1.16 | [1.00; 1.35] |
| | Sleep | pathological | 1.22 | [0.88; 1.70] | 1.18 | [0.93; 1.49] |
| | Social relationships | medium | 1.03 | [0.78; 1.35] | 0.79 | [0.62; 1.02] |
| | Social relationships | strong | 0.97 | [0.73; 1.28] | 0.74 | [0.57; 0.95] |
| | Sedentary behavior | | 0.99 | [0.97; 1.01] | 1.01 | [0.98; 1.03] |
| | Tobacco | occasional smoker | 0.86 | [0.67; 1.11] | 0.85 | [0.66; 1.10] |
| | Tobacco | daily smoker | 1.07 | [0.87; 1.31] | 1.05 | [0.87; 1.27] |
| | Alcohol | low risk | 1.70 | [1.32; 2.20] | 1.07 | [0.89; 1.28] |
| | Alcohol | minimal risk | 1.88 | [1.37; 2.57] | 1.21 | [0.96; 1.53] |
| | Alcohol | medium risk | 1.82 | [1.12; 2.95] | 0.94 | [0.64; 1.39] |
| | Alcohol | increased risk | 2.00 | [1.11; 3.60] | 1.54 | [0.76; 3.16] |
| | Drugs | >12 months ago | 1.13 | [0.96; 1.34] | 1.34 | [1.13; 1.58] |
| | Drugs | in the last 12 months | 0.87 | [0.60; 1.27] | 1.36 | [0.88; 2.10] |
| | Drugs | in the last 30 days | 0.96 | [0.65; 1.40] | 1.09 | [0.64; 1.86] |

| LBP | Variable | Parameter | Adjusted OR (male) | 95% CI (male) | Adjusted OR (female) | 95% CI (female) |
|---|---|---|---|---|---|---|
| | Depression | Mild depression symptoms | 1.42 | [1.17; 1.73] | 1.25 | [1.06; 1.48] |
| | Depression | Moderate depression symptoms | 1.60 | [1.07; 2.38] | 1.74 | [1.27; 2.39] |
| | Depression | Rather severe depression symptoms | 1.76 | [0.95; 3.28] | 1.26 | [0.74; 2.14] |
| | Depression | Severe depression symptoms | 2.24 | [0.82; 6.11] | 1.34 | [0.50; 3.56] |
| | Anxiety | Light | 1.28 | [1.03; 1.58] | 1.31 | [1.10; 1.56] |
| | Anxiety | Moderate | 1.74 | [1.10; 2.74] | 1.48 | [1.03; 2.14] |
| | Anxiety | Severe | 0.86 | [0.38; 1.91] | 0.83 | [0.47; 1.47] |
| | Arterial hypertension | Yes | 1.10 | [0.91; 1.32] | 1.17 | [0.97; 1.40] |
| | Hypercholesterolaemia | Yes | 0.93 | [0.77; 1.12] | 1.00 | [0.83; 1.22] |
| | Diabetes | Yes | 1.12 | [0.84; 1.49] | 0.84 | [0.60; 1.18] |
| | Age category | 18-24 years | 0.64 | [0.44; 0.92] | 1.10 | [0.82; 1.49] |
| | Age category | 25-34 years | 1.10 | [0.84; 1.45] | 1.10 | [0.87; 1.40] |
| | Age category | 35-44 years | 0.91 | [0.72; 1.14] | 0.92 | [0.75; 1.12] |
| | Age category | 55-64 years | 0.92 | [0.74; 1.13] | 0.92 | [0.76; 1.11] |
| | Age category | 65-74 years | 1.03 | [0.81; 1.30] | 0.87 | [0.71; 1.08] |
| | Age category | 75+ years | 0.96 | [0.70; 1.31] | 0.92 | [0.69; 1.24] |
| | BMI category | Underweight | 0.85 | [0.44; 1.67] | 0.97 | [0.74; 1.28] |
| | BMI category | Overweight | 0.81 | [0.69; 0.95] | 1.30 | [1.11; 1.51] |
| | BMI category | Obesity | 1.16 | [0.92; 1.45] | 1.25 | [1.01; 1.55] |
| | Civil status | Married | 0.97 | [0.80; 1.18] | 1.17 | [0.98; 1.40] |
| | Civil status | Divorced | 1.05 | [0.79; 1.4] | 1.27 | [1.00; 1.63] |
| | Civil status | Widowed | 1.29 | [0.73; 2.28] | 1.28 | [0.91; 1.81] |

| LBP | Variable | Parameter | Adjusted OR (male) | 95% CI (male) | Adjusted OR (female) | 95% CI (female) |
|---|---|---|---|---|---|---|
| | Nationality | Foreigner | 0.96 | [0.80; 1.15] | 1.03 | [0.87; 1.22] |
| | Language region | French-speaking Switzerland | 1.06 | [0.91; 1.25] | 1.08 | [0.93; 1.24] |
| | Language region | Italian-speaking Switzerland | 0.76 | [0.59; 0.97] | 0.93 | [0.76; 1.15] |
| | Education | Upper secondary education | 1.00 | [0.76; 1.32] | 0.92 | [0.74; 1.15] |
| | Education | Tertiary level | 0.77 | [0.57; 1.03] | 0.85 | [0.67; 1.07] |
| Strong LBP | Physical activity level | Partially active | 0.73 | [0.41; 1.32] | 0.81 | [0.54; 1.22] |
| | Physical activity level | Irregularly active | 0.66 | [0.38; 1.17] | 0.73 | [0.49; 1.09] |
| | Physical activity level | Regularly active | 0.77 | [0.43; 1.37] | 0.71 | [0.47; 1.09] |
| | Physical activity level | Trained | 0.60 | [0.33; 1.1] | 0.49 | [0.32; 0.75] |
| | Diet | 0-2 portions | 1.04 | [0.67; 1.6] | 0.85 | [0.54; 1.35] |
| | Diet | 3-4 portions | 1.12 | [0.69; 1.83] | 0.83 | [0.52; 1.32] |
| | Diet | ≥5 portions | 1.52 | [0.84; 2.73] | 0.99 | [0.60; 1.62] |
| | Sleep | moderate | 1.46 | [1.04; 2.05] | 1.98 | [1.54; 2.54] |
| | Sleep | pathological | 1.63 | [1.01; 2.63] | 3.53 | [2.51; 4.97] |
| | Social relationships | medium | 0.72 | [0.44; 1.17] | 0.60 | [0.41; 0.87] |
| | Social relationships | strong | 0.66 | [0.40; 1.10] | 0.52 | [0.35; 0.76] |
| | Sedentary behaviour | | 1.01 | [0.96; 1.06] | 1.01 | [0.97; 1.05] |
| | Tobacco | occasional smoker | 1.89 | [1.13; 3.15] | 1.24 | [0.78; 1.97] |
| | Tobacco | daily smoker | 2.07 | [1.43; 2.99] | 1.39 | [1.04; 1.86] |
| | Alcohol | low risk | 0.99 | [0.65; 1.51] | 0.89 | [0.67; 1.19] |
| | Alcohol | minimal risk | 0.98 | [0.53; 1.78] | 0.71 | [0.45; 1.12] |
| | Alcohol | medium risk | 0.43 | [0.16; 1.15] | 0.70 | [0.37; 1.300] |

| LBP | Variable | Parameter | Adjusted OR (male) | 95% CI (male) | Adjusted OR (female) | 95% CI (female) |
|---|---|---|---|---|---|---|
| | Alcohol | increased risk | 2.17 | [0.84; 5.60] | 0.51 | [0.12; 2.15] |
| | Drugs | >12 months ago | 1.02 | [0.72; 1.44] | 1.37 | [1.02; 1.83] |
| | Drugs | in the last 12 months | 1.25 | [0.60; 2.60] | 1.60 | [0.76; 3.35] |
| | Drugs | in the last 30 days | 1.24 | [0.62; 2.51] | 1.28 | [0.54; 2.99] |
| | Depression | Mild depression symptoms | 2.26 | [1.54; 3.31] | 1.52 | [1.12; 2.07] |
| | Depression | Moderate depression symptoms | 4.84 | [2.50; 9.35] | 2.25 | [1.40; 3.62] |
| | Depression | Rather severe depression symptoms | 4.75 | [2.09; 10.80] | 2.77 | [1.37; 5.61] |
| | Depression | Severe depression symptoms | 6.06 | [1.90; 19.26] | 1.62 | [0.51; 5.08] |
| | Anxiety | Light | 1.19 | [0.78; 1.82] | 1.52 | [1.12; 2.06] |
| | Anxiety | Moderate | 2.70 | [1.31; 5.55] | 1.61 | [0.97; 2.67] |
| | Anxiety | Severe | 1.10 | [0.41; 2.96] | 0.85 | [0.39; 1.86] |
| | Arterial hypertension | Yes | 1.48 | [1.05; 2.10] | 1.27 | [0.92; 1.74] |
| | Hypercholesterolaemia | Yes | 1.23 | [0.84; 1.79] | 1.16 | [0.83; 1.62] |
| | Diabetes | Yes | 1.23 | [0.77; 1.98] | 0.99 | [0.56; 1.76] |
| | Age category | 18-24 years | 0.33 | [0.13; 0.87] | 0.95 | [0.54; 1.68] |
| | Age category | 25-34 years | 0.90 | [0.49; 1.66] | 1.33 | [0.85; 2.08] |
| | Age category | 35-44 years | 0.77 | [0.46; 1.29] | 1.37 | [0.94; 1.98] |
| | Age category | 55-64 years | 1.09 | [0.70; 1.71] | 1.19 | [0.83; 1.71] |
| | Age category | 65-74 years | 1.30 | [0.78; 2.17] | 0.92 | [0.61; 1.40] |
| | Age category | 75+ years | 0.91 | [0.48; 1.73] | 1.24 | [0.71; 2.17] |
| | BMI category | Underweight | 1.62 | [0.42; 6.19] | 1.01 | [0.59; 1.71] |
| | BMI category | Overweight | 0.77 | [0.55; 1.07] | 1.21 | [0.92; 1.57] |

| LBP | Variable | Parameter | Adjusted OR (male) | 95% CI (male) | Adjusted OR (female) | 95% CI (female) |
|---|---|---|---|---|---|---|
| | BMI category | Obesity | 1.34 | [0.89; 1.99] | 1.62 | [1.15; 2.27] |
| | Civil status | Married | 1.98 | [1.32; 2.98] | 1.42 | [1.03; 1.97] |
| | Civil status | Divorced | 1.57 | [0.86; 2.88] | 1.87 | [1.21; 2.88] |
| | Civil status | Widowed | 1.32 | [0.38; 4.62] | 1.87 | [1.08; 3.24] |
| | Nationality | Foreigner | 1.22 | [0.83; 1.78] | 1.10 | [0.83; 1.45] |
| | Language region | French-speaking Switzerland | 0.95 | [0.69; 1.30] | 1.06 | [0.83; 1.35] |
| | Language region | Italian-speaking Switzerland | 0.61 | [0.35; 1.07] | 0.70 | [0.47; 1.03] |
| | Education | Upper secondary education | 2.72 | [1.44; 5.13] | 0.84 | [0.60; 1.19] |
| | Education | Tertiary level | 1.51 | [0.79; 2.89] | 0.53 | [0.36; 0.78] |
