# Supplementary material for: Leisure-time physical activity and low back pain in Switzerland: results from the 2022 cross-sectional Swiss Health Survey
Source: BMC Musculoskelet Disord. 2026 Jun 30;27:808. doi: 10.1186/s12891-026-10099-2 (PMC13621532; doi:10.1186/s12891-026-10099-2)
Supplement: Supplementary file 2 [file 12891_2026_10099_MOESM2_ESM.pdf]

Additional file 3. Descriptive (weighted) statistics for low back pain, separated by sex.

Characteristic	Male<br>N = 6,211	Female<br>N = 7,207	Overall<br>N = 13,418
**Age category**			
18-24 years	9.6%	9.1%	9.3%
25-34 years	18%	17%	17%
35-44 years	18%	19%	18%
45-54 years	18%	18%	18%
55-64 years	19%	18%	19%
65-74 years	13%	13%	13%
75+ years	4.7%	5.0%	4.9%
**BMI**			
Normal weight	46%	62%	54%
Underweight	1.1%	5.5%	3.3%
Overweight	40%	22%	30%
Obesity	13%	11%	12%
**Civil status**			
Single	38%	33%	36%
Married	52%	51%	51%
Divorced	8.6%	12%	10%
Widowed	1.2%	3.7%	2.5%
**Education**			
Compulsory school	7.7%	9.8%	8.8%
Upper secondary education	43%	49%	46%
Tertiary level	49%	42%	45%
**Nationality**			
Swiss	78%	79%	78%
Foreigner	22%	21%	22%
**Language regions of Switzerland**			
German-speaking part	73%	72%	73%
French-speaking part	23%	23%	23%
Italian-speaking part	4.2%	4.8%	4.5%
**Low back pain**			
No	60%	50%	55%
A little	34%	41%	38%
Strong	5.3%	8.7%	7.1%
**Low back pain - binary**	40%	50%	45%
**Physical activity category**			
Inactive	5.3%	6.3%	5.8%
Partially active	14%	17%	15%
Irregularly active	29%	30%	29%
Regularly active	16%	19%	18%
Trained	36%	28%	32%

Legend: Weighted mean %, Sum per column.
Abbreviation: BMI: Body Mass Index; CI: Confidence Interval.
