# Supplementary material for: Leisure-time physical activity and low back pain in Switzerland: results from the 2022 cross-sectional Swiss Health Survey
Source: BMC Musculoskelet Disord. 2026 Jun 30;27:808. doi: 10.1186/s12891-026-10099-2 (PMC13621532; doi:10.1186/s12891-026-10099-2)
Supplement: Supplementary file 3 [file 12891_2026_10099_MOESM3_ESM.pdf]

Additional file 4. Descriptive (weighted) statistics for physical activity, separated by sex.

	Male					Female					Overall				
**Characteristic**	**Inactive** N = 314	**Partially active** N = 875	**Irregularly active** N = 1,847	**Regularly active** N = 1,012	**Trained** N = 2,163	**Inactive** N = 444	**Partially active** N = 1,204	**Irregularly active** N = 2,146	**Regularly active** N = 1,418	**Trained** N = 1,995	**Inactive** N = 758	**Partially active** N = 2,079	**Irregularly active** N = 3,993	**Regularly active** N = 2,430	**Trained** N = 4,158
**Age**															
18-24 years	3.4%	9.1%	19%	14%	55%	5.2%	18%	25%	14%	38%	4.3%	14%	22%	14%	46%
25-34 years	3.2%	14%	27%	12%	45%	6.1%	18%	29%	19%	28%	4.6%	16%	28%	15%	37%
35-44 years	5.9%	18%	31%	16%	30%	5.7%	19%	29%	20%	27%	5.8%	18%	30%	18%	28%
45-54 years	7.8%	16%	28%	15%	33%	5.5%	18%	32%	18%	26%	6.6%	17%	30%	17%	29%
55-64 years	5.0%	15%	31%	17%	32%	6.8%	16%	32%	19%	26%	5.9%	16%	31%	18%	29%
65-74 years	5.1%	9.5%	32%	20%	34%	6.8%	13%	28%	22%	29%	5.9%	11%	30%	21%	32%
75+ years	6.7%	9.1%	32%	23%	28%	11%	14%	27%	29%	17%	9.2%	12%	30%	27%	23%
**BMI**															
Normal weight	4.4%	13%	25%	15%	42%	4.8%	16%	29%	20%	31%	4.6%	15%	27%	18%	36%
Underweight	4.4%	20%	23%	19%	33%	6.8%	18%	24%	22%	28%	6.5%	19%	24%	22%	29%
Overweight	5.3%	14%	32%	16%	34%	7.9%	19%	32%	17%	24%	6.2%	16%	32%	16%	30%
Obesity	8.2%	17%	33%	19%	23%	11%	19%	31%	21%	17%	9.7%	18%	32%	20%	20%
**Civil status**															
Single	5.2%	14%	25%	13%	42%	5.4%	19%	27%	17%	32%	5.3%	16%	26%	15%	37%
Married	4.6%	14%	32%	17%	32%	5.9%	17%	32%	20%	25%	5.2%	15%	32%	19%	29%
Divorced	9.0%	11%	26%	16%	38%	11%	15%	27%	21%	26%	9.9%	14%	27%	19%	31%
Widowed	7.4%	10%	31%	26%	25%	6.3%	13%	27%	24%	29%	6.6%	13%	28%	25%	28%
**Education**															
Compulsory school	13%	14%	22%	17%	34%	15%	18%	24%	21%	21%	14%	16%	23%	19%	26%
Upper secondary education	6.3%	14%	29%	17%	34%	6.2%	17%	30%	20%	27%	6.3%	15%	30%	19%	30%
Tertiary level	3.2%	14%	30%	15%	39%	4.3%	17%	30%	18%	31%	3.7%	15%	30%	16%	35%
**Nationality**															
Swiss	4.5%	14%	30%	16%	36%	4.8%	17%	30%	20%	28%	4.6%	16%	30%	18%	32%
Foreigner	8.0%	13%	26%	16%	37%	12%	17%	26%	18%	27%	9.9%	15%	26%	17%	32%
**Language regions of Switzerland**															
German-speaking part	4.9%	13%	30%	15%	37%	5.0%	15%	30%	20%	29%	4.9%	14%	30%	18%	33%
French-speaking part	6.6%	17%	25%	17%	34%	9.6%	23%	27%	16%	25%	8.2%	20%	26%	16%	30%
Italian-speaking part	5.1%	17%	29%	17%	32%	9.4%	19%	29%	22%	20%	7.4%	18%	29%	20%	25%
**Low back pain**															
No	4.8%	14%	28%	15%	39%	5.3%	16%	28%	20%	31%	5.0%	14%	28%	17%	35%
A little	4.8%	14%	31%	17%	33%	6.3%	18%	31%	19%	25%	5.7%	16%	31%	18%	29%
Strong	13%	16%	25%	16%	29%	12%	20%	28%	21%	19%	13%	18%	27%	19%	23%

Legend: Weighted %; Sum per row.
Abbreviations: BMI: Body Mass Index; N: Unweighted number of participants.
