# Supplementary material for: Leisure-time physical activity and low back pain in Switzerland: results from the 2022 cross-sectional Swiss Health Survey
Source: BMC Musculoskelet Disord. 2026 Jun 30;27:808. doi: 10.1186/s12891-026-10099-2 (PMC13621532; doi:10.1186/s12891-026-10099-2)
Supplement: Supplementary file 4 [file 12891_2026_10099_MOESM4_ESM.pdf]

Additional file 1. Outcome variables.

Outcome	Variable	Type of data collection	Swiss Health Survey 2022 variable	Abbreviations	Reference value	Other levels
Primary	LBP	Telephone	TKRSY01	lbp	No	A little, strong
	Physical activity	Index	ACTPHY5	pa_cat	Inactive	Partially active, irregularly active, regularly active, Trained
Secondary	Alcohol	Index	ALCCHRON5	alcohol	Abstinent	Low risk, minimal risk, medium risk, increased risk
	Anxiety	Index	ANXGAD	anxiety	None	Light, moderate, severe
	Arterial hypertension	Index	HYPERTENS	aht	No	Yes
	Depression	Index	DEPPHQ	dep	No/ minimal symptoms of depression	Mild depression symptoms, moderate depression symptoms, rather severe depressions symptoms, severe depression symptoms
	Diabetes mellitus	Index	DIABETE	diabetes	No	Yes
	Diet	Index	FIVEDAY	diet	<5 days per week	0-2 portions, 3-4 portions, ≥5 portions
	Drugs	Index	DROGCONS	drugs	Never used drugs	Never used cannabis, >12 months ago, in the last 12 months, in the last 30 days
	Hypercholesterolaemia	Index	HYPERCHOL	hyperchol	No	Yes
	Sedentary behaviour (numeric, hours per day)		TKOBW17	sed_beh	0	Numeric
	Sleeping disorder	Index	SOMMEIL	sleep	None or few	Moderate, pathological
	Social relationships/ Social support	Index	OSS3	soc	Low	Medium, strong
	Tobacco	Index	DAYSMOKE	tobacco	Non-smoker	Occasional smoker, daily smoker
Confounder	Age category	Telephone	ALTER7	age_cat	45-54 years	18-24 years, 25-34 years, 35-44 years, 55-64 years, 65-74 years, 75+ years
	BMI	Index	BMI4	bmi_cat	Normal weight	Underweight, overweight, obesity
	Civil status	Index	ETATCIV	civil_status	Single	Married, divorced, widowed
	Education	Index	AUSBILD3	education	Compulsory school	Upper secondary education, tertiary level

	Language region	Index	SPRACHE	lang_r	German-speaking Switzerland	French-speaking Switzerland, Italian-speaking Switzerland
	Nationality	Index	NATION2	nat	Swiss	Foreigner
	Sex	Telephone	SEX	sex	Male	Female

Legend: BMI: Body Mass Index; CHF: Swiss Francs.
