# Supplementary material for: Leisure-time physical activity and low back pain in Switzerland: results from the 2022 cross-sectional Swiss Health Survey
Source: BMC Musculoskelet Disord. 2026 Jun 30;27:808. doi: 10.1186/s12891-026-10099-2 (PMC13621532; doi:10.1186/s12891-026-10099-2)
Supplement: Supplementary file 5 [file 12891_2026_10099_MOESM5_ESM.pdf]

Additional file 2. Model formula.

Model 1: Unadjusted model for research question 1 (low back pain & physical activity)

$$\log\left(\frac{P(\,LBP_i = k \mid X_i\,)}{P(\,LBP_i = 0 \mid X_i\,)}\right) =$$

$$\beta_0^{(k)} + \beta_1^{(k)} \cdot \mathrm{PA}_{\mathrm{i,Partially}} + \beta_2^{(k)} \cdot \mathrm{PA}_{\mathrm{i,Irregular}} + \beta_3^{(k)} \cdot \mathrm{PA}_{\mathrm{i,Regular}} + \beta_4^{(k)} \cdot \mathrm{PA}_{\mathrm{i,Trained}}$$

Model 2: Adjusted model for research question 1 (low back pain & physical activity), including confounder, separated by sex

$$\log\left(\frac{P(\,LBP_i = k \mid X_i\,)}{P(\,LBP_i = 0 \mid X_i\,)}\right) = \beta_0^{(k)} + \beta_1^{(k)} \cdot \mathrm{PA}_{\mathrm{i,Partially}} + \beta_2^{(k)} \cdot \mathrm{PA}_{\mathrm{i,Irregular}} + \beta_3^{(k)} \cdot \mathrm{PA}_{\mathrm{i,Regular}}$$

$$+\beta_4^{(k)} \cdot \mathrm{PA}_{\mathrm{i,Trained}} + \beta_5^{(k)} \cdot \mathrm{Age}_{\mathrm{i,25\text{-}34}} + \beta_6^{(k)} \cdot \mathrm{Age}_{\mathrm{i,35\text{-}44}}$$

$$+\beta_7^{(k)} \cdot \mathrm{Age}_{\mathrm{i,45\text{-}54}} + \beta_8^{(k)} \cdot \mathrm{Age}_{\mathrm{i,55\text{-}64}} + \beta_9^{(k)} \cdot \mathrm{Age}_{\mathrm{i,65\text{-}74}} + \beta_{10}^{(k)} \cdot \mathrm{Age}_{\mathrm{i,75+}}$$

$$+\beta_{11}^{(k)} \cdot \mathrm{BMI}_{\mathrm{i,Underweight}} + \beta_{12}^{(k)} \cdot \mathrm{BMI}_{\mathrm{i,Overweight}} + \beta_{13}^{(k)} \cdot \mathrm{BMI}_{\mathrm{i,Obese}}$$

$$+\beta_{14}^{(k)} \cdot \mathrm{CivilStatus}_{\mathrm{i,Married}} + \beta_{15}^{(k)} \cdot \mathrm{CivilStatus}_{\mathrm{i,Divorced}} + \beta_{16}^{(k)} \cdot \mathrm{CivilStatus}_{\mathrm{i,Widowed}}$$

$$+\beta_{17}^{(k)} \cdot \mathrm{Nationality}_{\mathrm{i,Foreigner}} + \beta_{18}^{(k)} \cdot \mathrm{Region}_{\mathrm{i,French}}$$

$$+\beta_{19}^{(k)} \cdot \mathrm{Region}_{\mathrm{i,Italian}}$$

$$+\beta_{20}^{(k)} \cdot \mathrm{Education}_{\mathrm{i,UpperSec}} + \beta_{21}^{(k)} \cdot \mathrm{Education}_{\mathrm{i,Tertiary}}$$

Model 3: Adjusted model for research question 2 including lifestyle factors and confounder, separated by sex

$$\log\left(\frac{P(\,LBP_i = k \mid X_i\,)}{P(\,LBP_i = 0 \mid X_i\,)}\right) = \beta_0^{(k)} + \beta_1^{(k)} \cdot \mathrm{PA}_{\mathrm{i,Partially}} + \beta_2^{(k)} \cdot \mathrm{PA}_{\mathrm{i,Irregular}} + \beta_3^{(k)} \cdot \mathrm{PA}_{\mathrm{i,Regular}}$$

$$+\beta_4^{(k)} \cdot \mathrm{PA}_{\mathrm{i,Trained}} + \beta_5^{(k)} \cdot Diet_{\mathrm{i,0\text{-}2portions}} + \beta_6^{(k)} \cdot Diet_{\mathrm{i,3\text{-}4portions}} + \beta_7^{(k)} \cdot Diet_{\mathrm{i,\geq 5\ portions}}$$

$$+\beta_8^{(k)} \cdot Sleep_{\mathrm{i,moderate}} + \beta_9^{(k)} \cdot Sleep_{\mathrm{i,pathological}} + \beta_{10}^{(k)} \cdot Social\ relationships_{\mathrm{i,medium}}$$

$$+\beta_{11}^{(k)} \cdot Social\ relationships_{\mathrm{i,strong}} + \beta_{12}^{(k)} \cdot Sedentary\ behaviour_{\mathrm{i}}$$

$$+\beta_{13}^{(k)} \cdot \mathrm{Age}_{\mathrm{i,25\text{-}34}} + \beta_{14}^{(k)} \cdot \mathrm{Age}_{\mathrm{i,35\text{-}44}} + \beta_{15}^{(k)} \cdot \mathrm{Age}_{\mathrm{i,45\text{-}54}} + \beta_{16}^{(k)} \cdot \mathrm{Age}_{\mathrm{i,55\text{-}64}}$$

$$+\beta_{17}^{(k)} \cdot \mathrm{Age}_{\mathrm{i,65\text{-}74}} + \beta_{18}^{(k)} \cdot \mathrm{Age}_{\mathrm{i,75+}}$$

$$+\beta_{19}^{(k)} \cdot \mathrm{BMI}_{\mathrm{i,Underweight}} + \beta_{20}^{(k)} \cdot \mathrm{BMI}_{\mathrm{i,Overweight}} + \beta^{(k)} \cdot \mathrm{BMI}_{\mathrm{i,Obese}}$$

$$+\beta_{22}^{(k)} \cdot \mathrm{CivilStatus}_{\mathrm{i,Married}} + \beta_{23}^{(k)} \cdot \mathrm{CivilStatus}_{\mathrm{i,Divorced}} + \beta_{24}^{(k)} \cdot \mathrm{CivilStatus}_{\mathrm{i,Widowed}}$$

$$+\beta_{25}^{(k)} \cdot \mathrm{Nationality}_{\mathrm{i,Foreigner}}$$

$$+\beta_{26}^{(k)} \cdot \mathrm{Region}_{\mathrm{i,French}} + \beta_{27}^{(k)} \cdot \mathrm{Region}_{\mathrm{i,Italian}}$$

$$+\beta_{28}^{(k)} \cdot \mathrm{Education}_{\mathrm{i,UpperSec}} + \beta_{29}^{(k)} \cdot \mathrm{Education}_{\mathrm{i,Tertiary}}$$

Model 4: Adjusted model for research question 2 including lifestyle factors, variable of the Hartvigsen et al model and confounder, separated by sex

$$\log\left(\frac{P(LBP_i = k \mid X_i)}{P(LBP_i = 0 \mid X_i)}\right) = \beta_0^{(k)} + \beta_1^{(k)} \cdot \mathrm{PA}_{\mathrm{i,Partially}} + \beta_2^{(k)} \cdot \mathrm{PA}_{\mathrm{i,Irregular}} + \beta_3^{(k)} \cdot \mathrm{PA}_{\mathrm{i,Regular}}$$

$$+\beta_4^{(k)} \cdot \mathrm{PA}_{\mathrm{i,Trained}} + \beta_5^{(k)} \cdot Diet_{\mathrm{i,0\text{-}2portions}} + \beta_6^{(k)} \cdot Diet_{\mathrm{i,3\text{-}4portions}} + \beta_7^{(k)} \cdot Diet_{\mathrm{i,\geq 5\ portions}}$$

$$+\beta_8^{(k)} \cdot Sleep_{\mathrm{i,moderate}} + \beta_9^{(k)} \cdot Sleep_{\mathrm{i,pathological}} + \beta_{10}^{(k)} \cdot Social\ relationships_{\mathrm{i,medium}}$$

$$+\beta_{11}^{(k)} \cdot Social\ relationships_{\mathrm{i,strong}} + \beta_{12}^{(k)} \cdot Sedentary\ behaviour_{\mathrm{i}}$$

$$+\beta_{13}^{(k)} \cdot \mathrm{Tobacco}_{\mathrm{i,occasional\ smoker}} + \beta_{14}^{(k)} \cdot \mathrm{Tobaccol}_{\mathrm{i,daily\ smoker}}$$

$$+\beta_{15}^{(k)} \cdot \mathrm{Alcohol}_{\mathrm{i,low\ risk}} + \beta_{16}^{(k)} \cdot \mathrm{Alcohol}_{\mathrm{i,minimal\ risk}} + \beta_{17}^{(k)} \cdot \mathrm{Alcohol}_{\mathrm{i,medium\ risk}}$$

$$+\beta_{18}^{(k)} \cdot \mathrm{Alcohol}_{\mathrm{i,increased\ risk}} + \beta_{19}^{(k)} \cdot \mathrm{Drugs}_{\mathrm{i,never\ used\ cannabis}} + \beta_{20}^{(k)} \cdot \mathrm{Drugs}_{\mathrm{i,>12\ months\ ago}}$$

$$+\beta_{21}^{(k)} \cdot \mathrm{Drugs}_{\mathrm{i,in\ the\ last\ 12\ months}} + \beta_{22}^{(k)} \cdot \mathrm{Drugs}_{\mathrm{i,in\ the\ last\ 30\ days}}$$

$$+\beta_{23}^{(k)} \cdot \mathrm{Depression}_{\mathrm{i,mild\ depression\ symptoms}} + \beta_{24}^{(k)} \cdot \mathrm{Depression}_{\mathrm{i,moderate\ depression\ symptoms}}$$

$$+\beta_{25}^{(k)} \cdot \mathrm{Depression}_{\mathrm{i,rather\ severe\ depression\ symptoms}} + \beta_{26}^{(k)} \cdot \mathrm{Depression}_{\mathrm{i,severe\ depression\ symptoms}}$$

$$+\beta_{27}^{(k)} \cdot \mathrm{Anxiety}_{\mathrm{i,light}} + \beta_{28}^{(k)} \cdot \mathrm{Anxiety}_{\mathrm{i,moderate}} + \beta_{29}^{(k)} \cdot \mathrm{Anxiety}_{\mathrm{i,severe}}$$

$$+\beta_{30}^{(k)} \cdot \mathrm{Art.Hypertension}_{\mathrm{i,yes}} + \beta_{31}^{(k)} \cdot \mathrm{Hypercholesterolaemia}_{\mathrm{i,yes}} + \beta_{32}^{(k)} \cdot \mathrm{Diabetes}_{\mathrm{i,yes}}$$

$$+\beta_{33}^{(k)} \cdot \mathrm{Age}_{\mathrm{i,25\text{-}34}} + \beta_{34}^{(k)} \cdot \mathrm{Age}_{\mathrm{i,35\text{-}44}} + \beta_{35}^{(k)} \cdot \mathrm{Age}_{\mathrm{i,45\text{-}54}} + \beta_{36}^{(k)} \cdot \mathrm{Age}_{\mathrm{i,55\text{-}64}}$$

$$+\beta_{37}^{(k)} \cdot \mathrm{Age}_{\mathrm{i,65\text{-}74}} + \beta_{38}^{(k)} \cdot \mathrm{Age}_{\mathrm{i,75+}}$$

$$+\beta_{39}^{(k)} \cdot \mathrm{BMI}_{\mathrm{i,Underweight}} + \beta_{40}^{(k)} \cdot \mathrm{BMI}_{\mathrm{i,Overweight}} + \beta_{41}^{(k)} \cdot \mathrm{BMI}_{\mathrm{i,Obese}}$$

$$+\beta_{42}^{(k)} \cdot \mathrm{CivilStatus}_{\mathrm{i,Married}} + \beta_{43}^{(k)} \cdot \mathrm{CivilStatus}_{\mathrm{i,Divorced}} + \beta_{44}^{(k)} \cdot \mathrm{CivilStatus}_{\mathrm{i,Widowed}}$$

$$+\beta_{45}^{(k)} \cdot \mathrm{Nationality}_{\mathrm{i,Foreigner}}$$

$$+\beta_{46}^{(k)} \cdot \mathrm{Region}_{\mathrm{i,French}} + \beta_{47}^{(k)} \cdot \mathrm{Region}_{\mathrm{i,Italian}}$$

$$+\beta_{48}^{(k)} \cdot \mathrm{Education}_{\mathrm{i,UpperSec}} + \beta_{49}^{(k)} \cdot \mathrm{Education}_{\mathrm{i,Tertiary}}$$

The abbreviations stand for:

- $P(LBP_i = k \mid X)$ represents the probability that an individual *i* belongs to back pain category *k* for a given set of X.
- *k* = 0,1,2 represent the back pain levels (No pain, A litte, Strong)..
- *X* represents the set of predictors, e.g. physical activity.
- $\beta_{j,k}$ are the regression coefficients for predictor *j* and category *k*.
- ϵ_i represents the error term for individual *i*.
